# Identification of biomarkers and construction of discriminating model for tuberculosis patients with diabetes mellitus based on proteomics: a cross-sectional study

**DOI:** 10.3389/fimmu.2025.1713654

**Published:** 2026-01-15

**Authors:** Yufeng Li, Peng Cheng, Yajing An, Ruizi Ni, Zhaoyang Ye, Ling Yang, Li Zhuang, Linsheng Li, Liang Wang, Wenping Gong

**Affiliations:** 1 Senior Department of Tuberculosis, Chinese PLA General Hospital, Beijing, China; 2 Graduate School, Hebei North University, Zhangjiakou, Hebei, China; 3 Department of Geriatrics, the Eighth Medical Center of PLA General Hospital, Beijing, China; 4 Handan Municipal Centre for Disease Prevention and Control, Handan, Hebei, China

**Keywords:** cytokines, diabetes mellitus, lymphocyte subsets, proteomics, tuberculosis, tuberculosis-diabetes comorbidity (TB-DM)

## Abstract

**Background:**

Tuberculosis-diabetes mellitus (TB-DM) comorbidity presents significant clinical challenges due to poor treatment outcomes. This study investigated peripheral blood lymphocyte profiles and cytokine dynamics in TB-DM patients compared to healthy controls (HCs) and DM patients.

**Methods:**

Subjects from the healthy controls (HCs), DM, and TB-DM were recruited, and peripheral blood samples were collected. The absolute counts of lymphocyte subsets were detected by flow cytometry, and the cytokines were quantitatively analyzed using the Olink ultra-sensitive targeted protein detection technology for micro-samples. Methods such as differential expression analysis, principal component analysis (PCA), correlation analysis, KEGG pathway enrichment analysis, and GO functional annotation were used to screen out the biomarkers related to TB-DM. Based on this, a TB-DM internal model performance was constructed, and the receiver operating characteristic (ROC) curve was used to evaluate its diagnostic efficacy.

**Results:**

The study demonstrated significantly reduced NK cells (P_TB-DM vs. HC_ < 0.0001 and P_TB-DM vs. DM_ = 0.0292), total T cells (P_TB-DM vs. HC_ = 0.0018 and P_TB-DM vs. DM_ < 0.0001), and CD8+ T cells (P_TB-DM vs. HC_ = 0.0009 and P_TB-DM vs. DM_ = 0.0072) in TB-DM versus HCs and DM groups. TB-DM patients showed decreased CD4+ T (P_TB-DM vs. DM_ < 0.0001) and B cells (P_TB-DM vs. DM_ = 0.0004) compared to DM controls. Cytokine profiling revealed 5 upregulated and 17 downregulated factors in TB-DM. Three biomarkers (IL-6, IFN-γ, CXCL10) demonstrated superior diagnostic performance (AUC = 0.9841, sensitivity=88.89%, specificity=92.86%) when combined.

**Conclusion:**

Our findings identify distinct immunological alterations in TB-DM and propose a novel cytokine-based diagnostic panel for this high-risk population.

## 1 Introduction

Tuberculosis (TB), a chronic respiratory infectious disease caused by *Mycobacterium tuberculosis* (MTB), remains a critical global health challenge (1, 2). According to the World Health Organization (WHO) 2025 Global Tuberculosis Report, an estimated 10.7 million individuals developed TB worldwide in 2024, with 1.23 million deaths, maintaining its status as the leading cause of mortality from a single infectious pathogen (3). Concurrently, diabetes mellitus (DM), the fastest-growing endocrine metabolic disorder, presents a substantial epidemiological burden. The International Diabetes Federation (IDF) reported 589 million adults aged 20–79 living with DM in 2021, projected to rise to 853 million by 2050 (4). Notably, the bidirectional interaction between these two diseases has emerged as a major public health concern (5–8).

Accumulating evidence demonstrates a synergistic detrimental relationship between DM and TB (9–11). DM increases the risk of active tuberculosis (ATB) development by 2-4-fold, establishing it as a critical metabolic risk factor for TB (8, 12). A meta-analysis encompassing 22,658 studies revealed a global prevalence of 13.73% for TB-DM comorbidity, rising to 14.62% in high-burden regions such as Southeast Asia (13). DM compromises host immunity, exacerbating susceptibility to ATB, while TB-induced stress hyperglycemia further disrupts glycemic control (14, 15). Radiological analyses indicate that tuberculosis-diabetes comorbidity (TB-DM) patients exhibit higher rates of pulmonary cavitation on chest X-rays and increased incidence of adverse drug reactions during anti-TB therapy (16, 17). Importantly, DM significantly elevates treatment failure rates, mortality, and relapse rates in TB patients compared to non-diabetic counterparts (18). These findings underscore the profound impact of DM on TB pathogenesis, therapeutic outcomes, and prognosis (8).

Despite advances in understanding TB-DM interactions, critical knowledge gaps persist. Current diagnostic frameworks lack validated biomarkers for early comorbidity detection, and systematic characterization of immunometabolic alterations in TB-DM patients remains incomplete. This study employs flow cytometry and proteomic profiling to elucidate cellular and molecular immune signatures in TB-DM cohorts. Our objectives are to construct a TB-DM-specific immune profile and identify diagnostic biomarkers with clinical utility. These findings will establish novel immunological monitoring parameters and therapeutic strategies, and thereby aims to facilitate the early and accurate identification of tuberculosis co-infection in individuals with diabetes, ultimately improving clinical outcomes for TB-DM patients.

## 2 Materials and methods

### 2.1 Study design and ethical statement

This cross-sectional study was conducted at the Eighth Medical Center of PLA General Hospital from March to November 2022. Two independent population cohorts were enrolled, each comprising three subgroups: healthy controls (HCs), DM, and TB-DM patients. Inclusion criteria for HC included no history of tuberculosis exposure or infection, normal blood glucose levels, unremarkable chest X-ray findings, HIV-negative status, and age ≥18 years. Exclusion criteria consisted of: identifiable additional risk of TB exposure (such as occupation in TB care, close contact with ATB patients, or recent residence in or travel to high−incidence areas within China), prior history of TB or residual pulmonary lesions, contraindications to interferon−γ release assay (IGRA), and diagnosis of DM or HIV infection.

DM participants met the American Diabetes Association (ADA) diagnostic criteria (fasting blood glucose ≥7.0 mmol/L, or 2−h postprandial glucose ≥11.1 mmol/L, or HbA1c ≥6.5%, or random plasma glucose ≥11.1 mmol/L in the presence of classic hyperglycemic symptoms or hyperglycemic crisis) (19). Individuals with HIV infection, agranulocytosis, autoimmune diseases, severe hepatic or renal dysfunction, malignancy, immunomodulatory therapy, or pregnancy were excluded.

TB−DM participants fulfilled both the diagnostic criteria for DM and for ATB. All patients enrolled in the TB−DM group were required to have a documented history of DM, recorded by a formal medical institution prior to the current ATB diagnosis. This medical history was verified through the electronic medical record system. ATB was confirmed per Diagnostic Criteria for Pulmonary Tuberculosis (WS288-2017), requiring microbiological evidence (e.g., sputum smear positivity, mycobacterial culture, or nucleic acid detection) alongside clinical, radiological, and epidemiological findings. Enrolled TB-DM patients were aged 18–60 years, diagnosed with ATB ≤2 weeks without prior anti-tuberculosis therapy. Exclusion criteria included corticosteroid use, immunocompromised status (e.g., HIV, organ transplantation, autoimmune disorders), malnutrition, or ATB diagnosis >2 weeks with prior treatment.

Ethical approval was obtained from the Ethics Committee of the Eighth Medical Center of PLA General Hospital (Approval No. 309202204080808), with written informed consent from all participants.

### 2.2 Lymphocyte subset analysis

In Cohort I, 5 mL peripheral blood was collected per participant for lymphocyte profiling. Absolute counts of B cells, NK cells, NKT cells, total T cells, CD4^+^; T cells, and CD8^+^; T cells were quantified using 50 μL whole blood via flow cytometry. Reverse pipetting technique was employed to transfer 50 μL anticoagulated whole blood into Trucount™ tubes preloaded with 10 μL Multitest™ 6-color TBNK reagent. After vortex mixing and 15-minute dark incubation at room temperature, 450 μL lysing solution was added, followed by 15-minute incubation. Samples were analyzed using a FACSAria II flow cytometer.

### 2.3 Olink ultra-sensitive targeted protein detection for micro-samples

For Cohort II, 10 mL of the patient’s whole blood sample was centrifuged at 3000 rpm for 3–5 minutes, extracted the upper-layer plasma, and stored it at -80°C for future use. According to the manufacturer’s instructions, the Olink Target 96 Inflammation Panel is used to quantify the serum protein levels. Olink’s unique Proximity Extension Assay (PEA) provides a unique and efficient tool for the discovery and development of protein biomarkers. Antibody pairs labeled with DNA oligonucleotides specifically bind to the target antigens in solution. Through DNA polymerase, the adjacent DNA oligonucleotide chains hybridize and extend. The newly generated DNA barcodes are amplified by standard polymerase chain reaction (PCR). The amplified samples are transferred to an integrated microfluidic chip (IFC), loaded into the instrument for quantitative polymerase chain reaction (qPCR), and the raw data are generated (20). The bcl files generated by the qPCR detection are converted into counts files, and the NPX Manager software is used for quality control and data normalization. The final measurement results are expressed as the normalized protein expression (NPX).

### 2.4 Bioinformatics analysis

The R package “Olink Analyze” was employed to identify differentially expressed proteins (DEPs) between groups, using a statistical threshold of |log_2_ fold change| ≥ 1 and a P value < 0.05. We used the “ggplot2” package to construct a volcano plot to visualize the analysis results of these DEPs. The Kyoto Encyclopedia of Genes and Genomes (KEGG) online database and the Gene Ontology (GO) database were employed to conduct KEGG and GO enrichment analyses on the DEPs. The KEGG database helps to understand the advanced functions and utilities of biological systems (such as cells, organisms, and ecosystems) at the molecular level, especially from the large-scale molecular data generated by genomic sequencing and other high-throughput experimental techniques (21). The GO database is one of the most commonly used gene annotation databases. With the annotations in the GO database, functional enrichment analysis of gene sets can be carried out (22). The GO database is mainly divided into three parts, which describe the molecular function (MF), cellular component (CC), and biological process (BP) of genes respectively (23).

### 2.5 Statistical analysis

Statistical analyses were performed using GraphPad Prism (version 10.4.1) and SPSS (version 27.0). For immune cell counts and cytokine expression levels within a one−factor multi−level design, data distribution was first assessed. The normality of each group was tested individually using the Shapiro−Wilk test, and homogeneity of variances was verified using the Brown−Forsythe test. If all groups satisfied both normality and homoscedasticity, one−way analysis of variance (ANOVA) was applied, followed by Tukey’s post−hoc test for multiple comparisons when the overall ANOVA was significant. Otherwise, the non−parametric Kruskal−Wallis test was used, with Dunn’s test for post−hoc comparisons following a significant result.

Principal component analysis (PCA) was performed to evaluate variance contributions. Associations between variables were examined using Pearson correlation analysis, and significant correlations were further modeled by linear regression (reported with R² values and regression equations). The diagnostic efficacy of individual TB−DM biomarkers was evaluated by receiver operating characteristic (ROC) curve analysis, with confidence intervals calculated using the Wilson/Brown method. Additionally, composite biomarker probabilities derived from logistic regression models were validated using ROC analysis. A p−value < 0.05 was considered statistically significant.

## 3 Results

### 3.1 General characteristics of the included cases

**Figure 1:**
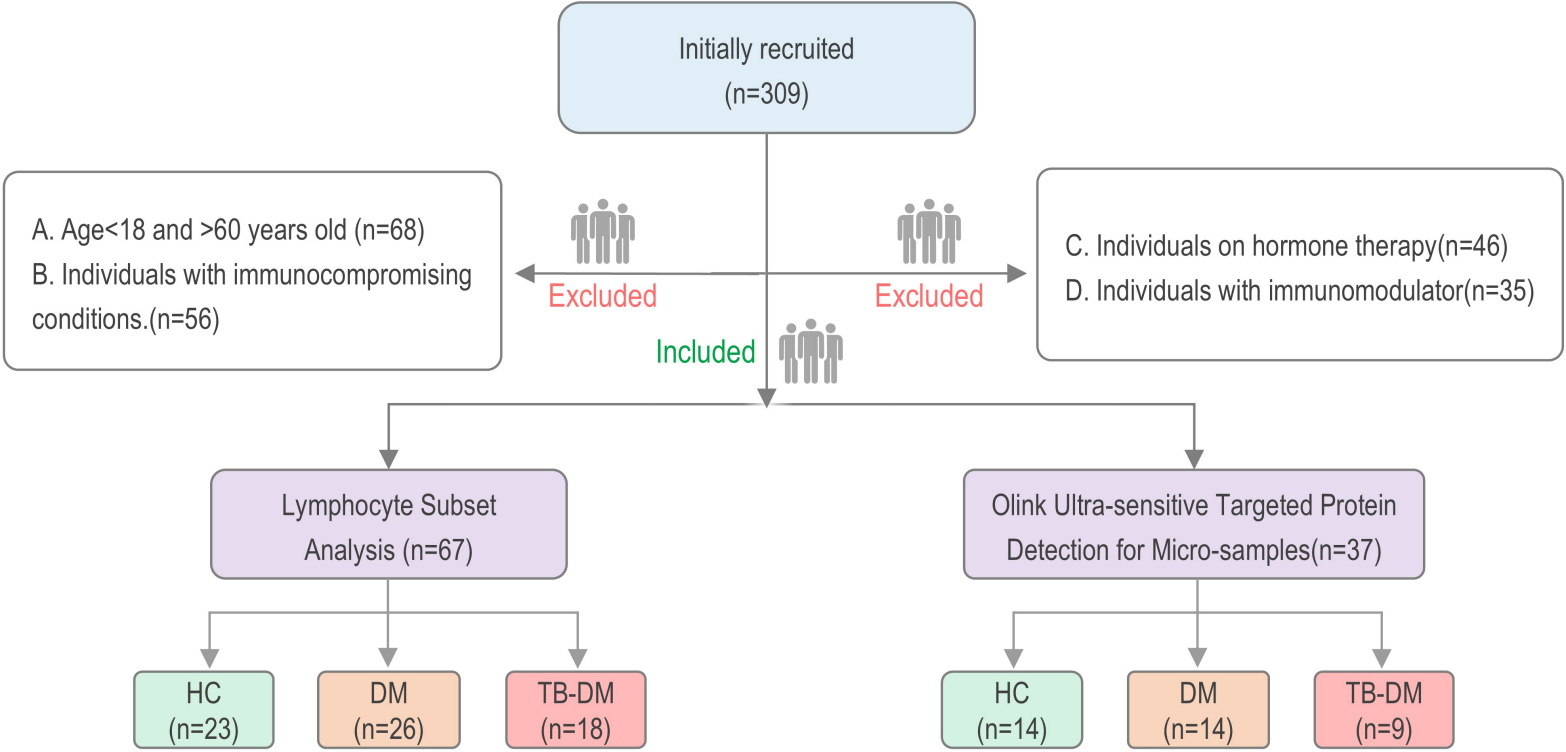
Participant Recruitment and Inclusion Process. A total of 309 participants were initially enrolled in this study. Based on the predefined inclusion and exclusion criteria, the following individuals were excluded: those younger than 18 or older than 60 years of age (n = 68), individuals with immunocompromising conditions (n = 56), individuals on hormone therapy (n = 46), and individuals receiving immunomodulatory treatment (n = 35). Consequently, 104 participants were ultimately included in the final analysis.

A total of 104 cases were enrolled in this study, divided into two cohorts: cohort I (67 cases) and cohort II (37 cases) (**Figure 1**). Cohort I included 23 HCs, 26 DM, and 18 TB-DM patients; gender distribution was male, 52 cases (77.6%), female, 15 cases (22.4%); age distributions were 35.74 ± 12.39 years for HCs, 46.81 ± 11.09 years for DM, and 52.44 ± 14.53 years for TB-DM. Cohort II included 14 HCs, 14 DM, and 9 TB-DM cases; gender distribution was male, 30 cases (81.1%), female, 7 cases (18.9%); age distributions were 39.07 ± 13.21 years for HCs, 43.57 ± 11.27 years for DM, and 51.78 + 15.79 years for TB-DM. Detailed baseline characteristics for each cohort are presented in **Supplementary Tables S1** and **S2**.

### 3.2 Quantitative analysis of immune cell subpopulations unveils severe imbalances in TB-DM patients

**Figure 2:**
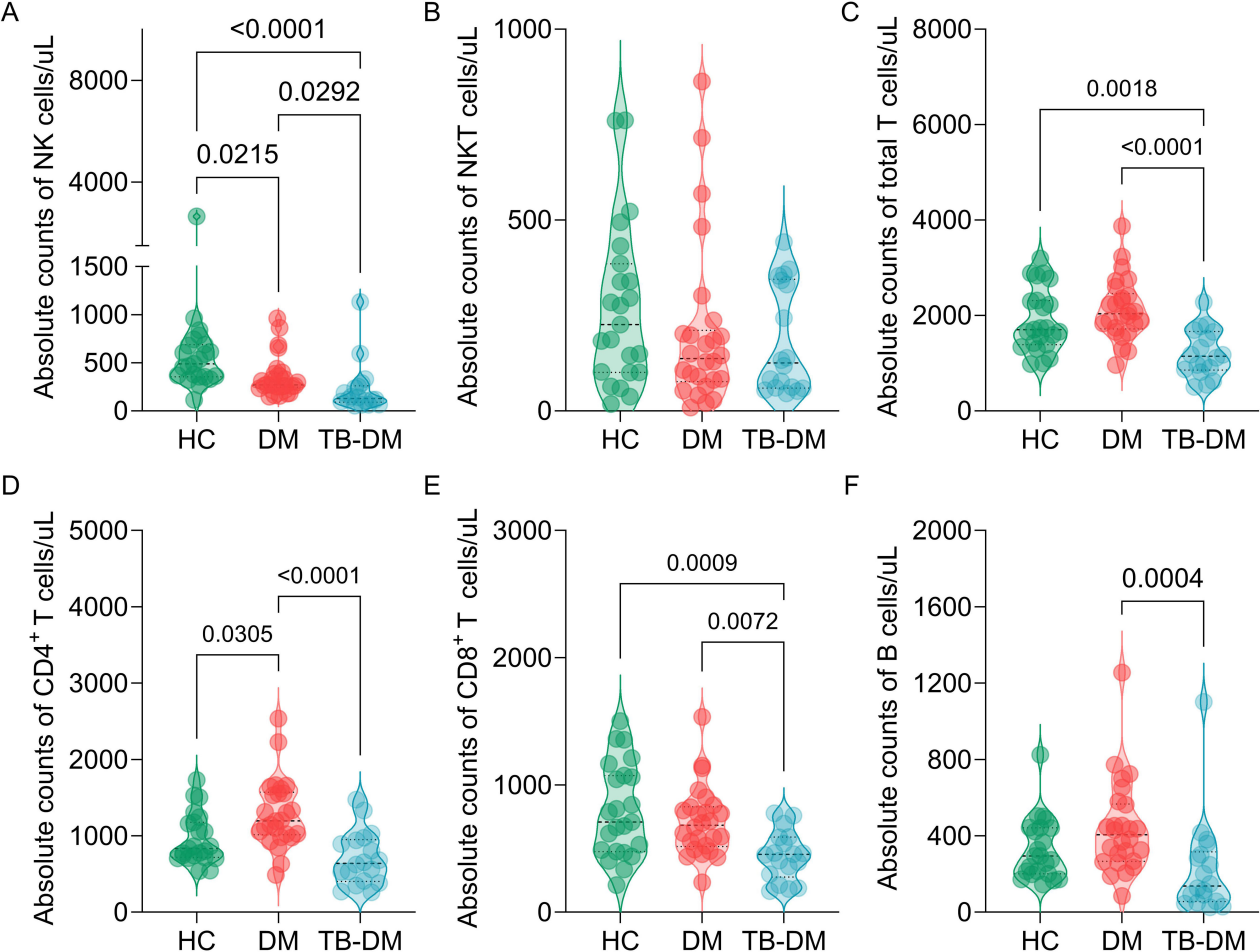
Comparative analysis of lymphocyte subsets across study groups. Absolute counts of lymphocyte populations in HCs, DM patients, and TB-DM comorbid patients. **(A)** NK cells. **(B)** Natural killer T (NKT) cells. **(C)** Total T lymphocytes. **(D)** CD4^+^; T lymphocytes. **(E)** CD8^+^; T lymphocytes. **(F)** B lymphocytes. Statistical significance was determined by one-way ANOVA or Kruskal-Wallis tests, depending on normality and variance homogeneity. Statistical significance was set at p<0.05.

NK cells showed a gradient decline in absolute counts, with HCs significantly higher than DM (P = 0.0215) and DM significantly higher than TB-DM (P = 0.0292). HCs showed a significant difference from TB-DM (P<0.0001) compared to DM (P = 0.0292) (**Figure 2A**). NKT cells showed no significant differences in absolute counts across the three groups (**Figure 2B**). Further analysis of adaptive immune cells revealed that TB-DM group had significantly lower total T cells (P = 0.0018) and CD8^+^ T cells (P = 0.0009) compared to HCs (P = 0.0018 for total T cells and P = 0.0009 for CD8^+^ T cells). Additionally, TB-DM group showed significantly lower total T cells (P<0.0001) and CD8^+^ T cells (P = 0.0072) compared to DM (P<0.0001 for total T cells and P = 0.0072 for CD8^+^ T cells). DM group also showed significantly higher CD4^+^ T cells compared to HCs (P = 0.0305) (**Figures 2C–E**). Furthermore, TB-DM group showed significantly lower B cells compared to DM (P = 0.0004) (**Figure 2F**).

### 3.3 Olink-based profiling reveals dysregulated cytokines in tuberculosis comorbid with diabetes mellitus

**Figure 3:**
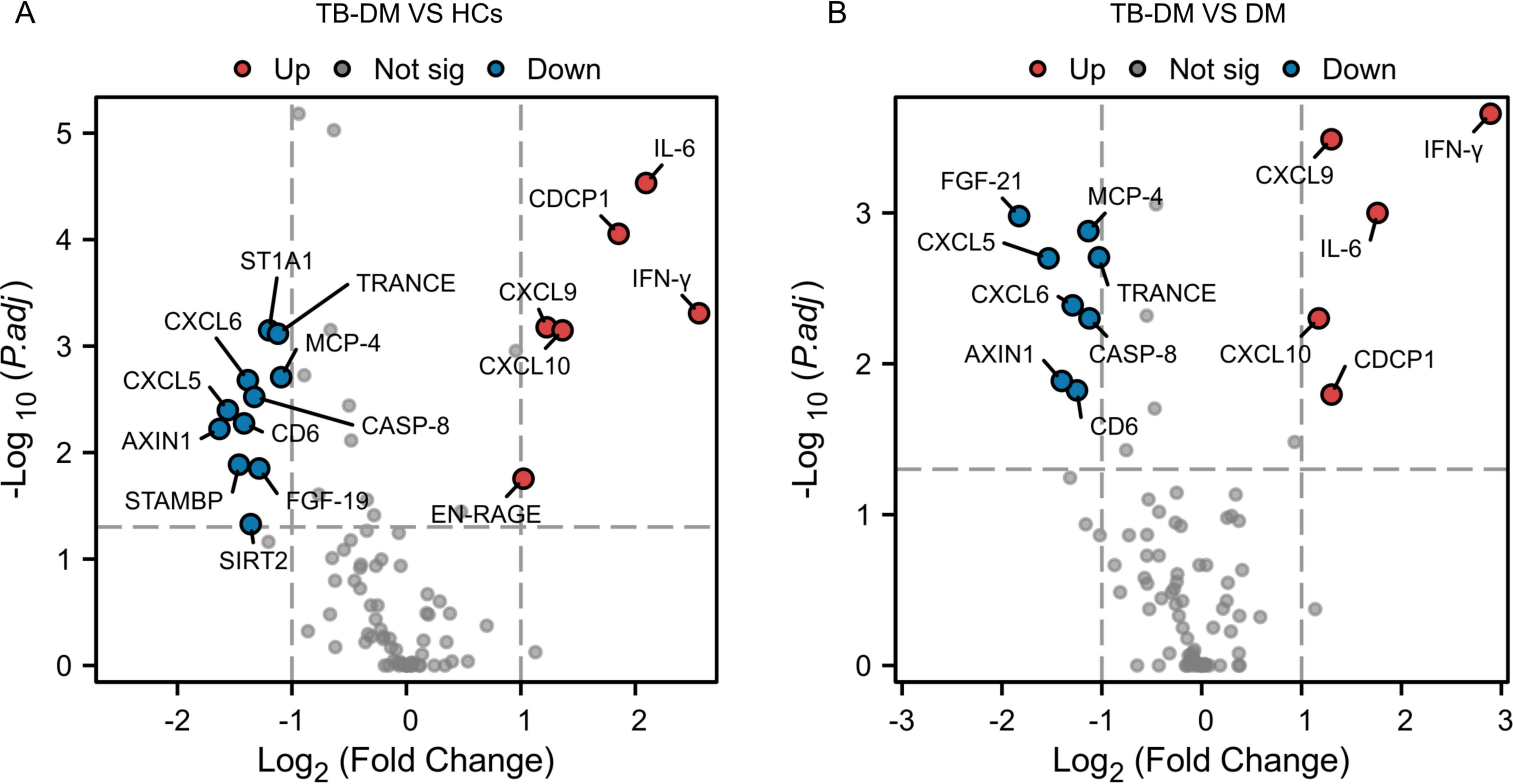
Volcano plots of differentially expressed cytokines. Differential cytokine expression analysis comparing: **(A)** TB-DM versus HCs (fold change >2.0, adjusted P <0.05); **(B)** TB-DM versus DM (fold change >2, adjusted P <0.05). Red dots: upregulated proteins; blue dots: downregulated proteins; gray dots: non-significant changes. Dashed lines indicate significance thresholds.

Using the Olink ultra-sensitive targeted protein detection technology for micro-samples, 92 cytokines were analyzed. Volcano plots revealed that compared to HCs, the TB-DM group exhibited 6 significantly upregulated cytokines, including interferon-gamma (IFN-γ), interleukin-6 (IL-6), C-X-C motif chemokine ligand 9 (CXCL9), CUB domain-containing protein 1 (CDCP1), CXCL10, and extracellular newly identified receptor for advanced glycation end-products (EN-RAGE), along with 11 significantly downregulated cytokines, namely sulfotransferase family 1A member 1 (ST1A1), TNF-related activation-induced cytokine (TRANCE), CXCL5, CXCL6, monocyte chemoattractant protein-4 (MCP-4), caspase-8 (CASP-8), Axis inhibition protein 1 (AXIN1), cluster of differentiation 6 (CD6), STAM-binding protein (STAMBP), sirtuin 2 (SIRT2), and Fibroblast growth factor 19 (FGF-19) (**Figure 3A**). Compared to DM group, the TB-DM group exhibited 5 significantly upregulated cytokines, which were IFN-γ, IL-6, CXCL9, CDCP1, and CXCL10, as well as 8 significantly downregulated cytokines, including FGF-21, MCP-4, CXCL5, CXCL6, TRANCE, CASP-8, AXIN1, and CD6 (**Figure 3B**).

### 3.4 Cytokine Expression Imbalances Among the Three Groups.

**Figure 4:**
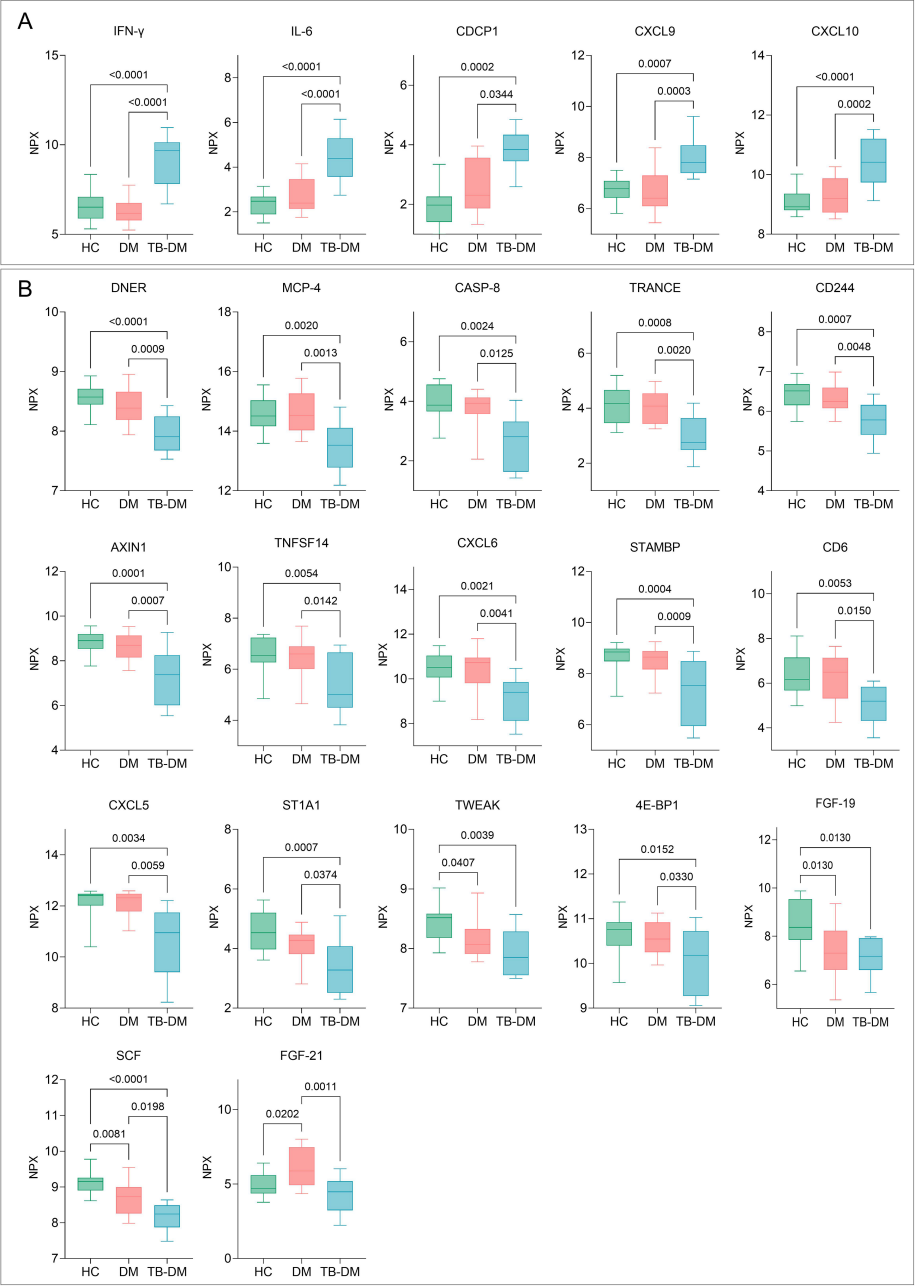
Cytokine profile alterations in TB-DM patients. **(A)** Five cytokines significantly elevated in TB-DM compared to both HCs and DM groups. **(B)** Seventeen cytokines showing marked reduction in TB-DM cohort. NPX (Normalized Protein Expression) values represent log2-transformed protein concentrations measured by Olink. Statistical significance was determined by one-way ANOVA or Kruskal-Wallis tests, depending on normality and variance homogeneity. Statistical significance was set at p<0.05.

Further analysis of cytokine expression differences among the three groups revealed that 22 cytokines exhibited expression differences in two groups. Compared to HCs and DM, the TB-DM group showed significantly higher IFN-γ, IL-6, CDCP1, CXCL9, and CXCL10 expression (**Figure 4A**). The TB-DM group also showed significant decreases in Delta/Notch-like EGF repeat-containing (DNER), MCP-4, CASP-8, TRANCE, and CD244 molecule (CD244) expression (**Figure 4B**). SCF expression showed a gradual decline with advancing disease progression and exhibited significant differences (**Figure 4B**). Additionally, FGF-21 expression was significantly higher in DM patients compared to HCs and TB-DM patients (**Figure 4B**).

### 3.5 Insights from PCA, correlation matrix analysis and linear regression analysis in different groups

**Figure 5:**
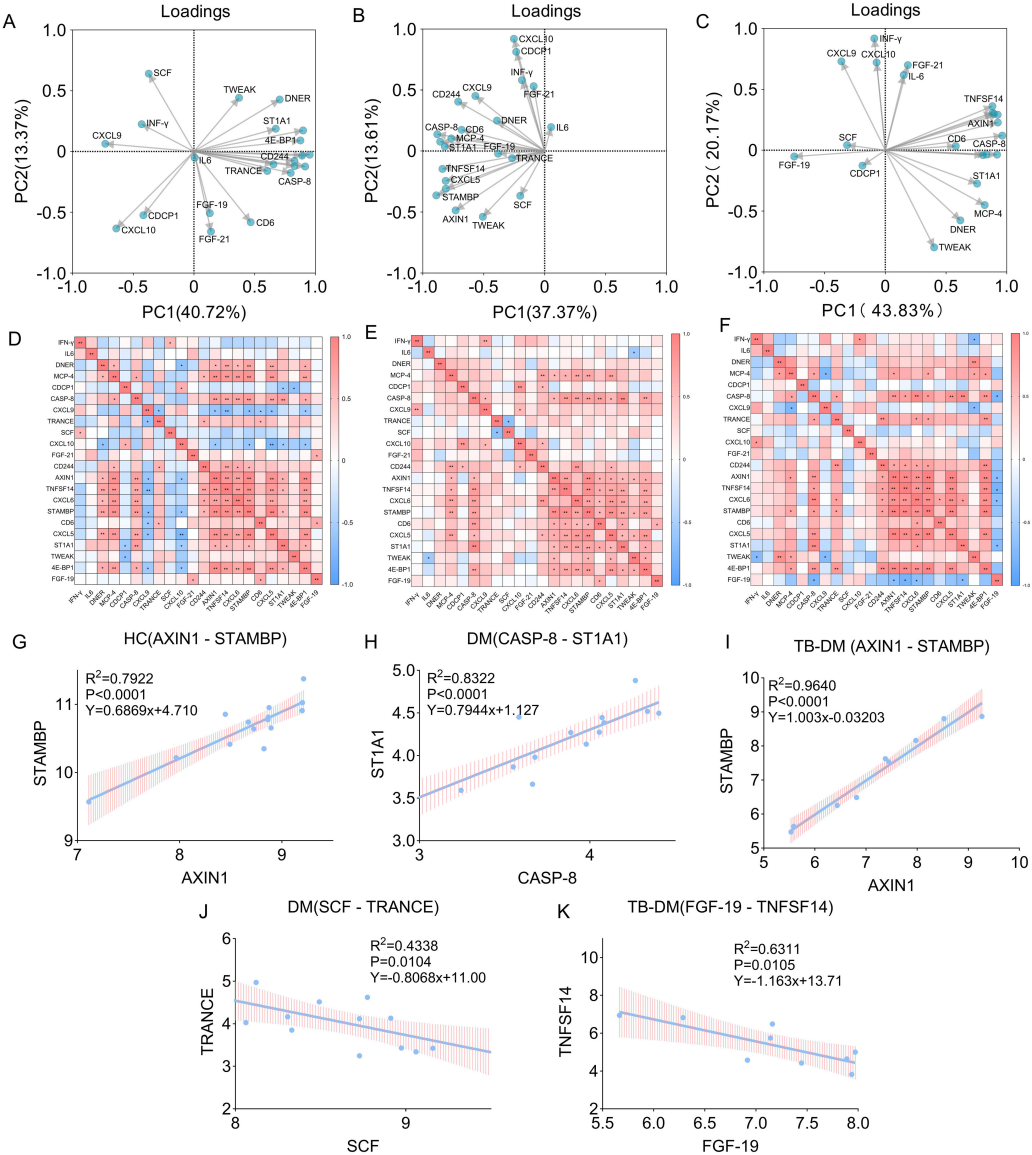
Multidimensional analysis of cytokine interactions. **(A–C)** Principal component analysis (PCA) score plots for HCs, DM, and TB-DM groups. Ellipses denote 95% confidence intervals. **(D–F)** Spearman correlation matrices (P <0.05). Red/blue hues indicate positive/negative correlations respectively, with intensity proportional to strength. **(G–K)** Univariate linear regression models of top-correlated cytokine pairs: **(G)** HCs: AXIN1 vs STAMBP (R²=0.7922). **(H)** DM: CASP-8 vs ST1A1 (R²=0.8322). **(I)** TB-DM: AXIN1 vs STAMBP (R²=0.9640). **(J, K)** Inverse correlations in DM (SCF vs TRANCE, R²=0.4338) and TB-DM (FGF-19 vs TNFSF14, R²=0.6311). P values: *<0.05, **<0.01.

To systematically depict the peripheral immune-regulatory landscape across HC, DM and TB-DM, we performed PCA (**Figures 5A–C**), Pearson correlation matrices (**Tables 1**, **2**; **Figures 5D–F**) and univariate linear regression of the top-correlated cytokine pairs (**Figures 5G–K**). The data reveal a stepwise remodelling of the cytokine covariance architecture that follows an “equilibrium–loosening–polarization” trajectory (**Figures 5A–C**). In HC, PC1 and PC2 jointly account for 54.09% of the variance, with SCF, IFN-γ, CXCL9, CXCL10 and CDCP1 clustering separately from the remaining IL-6-containing set (**Figure 5A**); this bipartite structure mirrors the balanced interplay shown in **Figure 5D**, where a positive backbone [AXIN1–TNFSF14 (Tumor Necrosis Factor Superfamily Member 14), CXCL6–STAMBP (STAM Binding Protein), etc.] is counter-weighted by widespread negative correlations for CXCL9/CXCL10. Linear modelling further identifies a strong positive AXIN1–STAMBP relationship (R² = 0.7922, P < 0.0001; **Figure 5G**), indicating that homeostasis is maintained by multiple co-equal covariance axes. Upon progression to DM, FGF-21 dissociates from its original cluster and forms a novel “metabo-inflammatory” module together with IFN-γ, IL-6, CXCL10 and CDCP1, while CXCL9 merges into the other major cluster; the combined PC1 + PC2 variance drops to 50.98% (**Figure 5B**), signifying initial coupling of hyperglycaemia-driven metabolic signals with Th1 pathways. **Figure 5E** captures the accompanying immune-regulatory shift: IL-6 attains a negative correlation with TNF-like weak inducer of apoptosis (TWEAK) (r = −0.56, P = 0.037) and SCF–TRANCE exhibits a significant inverse regression (R² = 0.4338, P = 0.0104; **Figure 5J**), marking the first emergence of immunosuppressive cues. Once TB-DM develops, PCA resolves into three polarized clusters: FGF-21, IFN-γ, IL-6, CXCL9 and CXCL10 constitute an “inflammatory-metabolic” pole, whereas negative PC scores aggregate negative regulators such as TWEAK, FGF-19 and CASP-8; PC1 + PC2 variance now surges to 64.00% (**Figure 5C**), implying dominance by a few robust covariance axes. Network fragmentation becomes even more pronounced in **Figure 5F**: the AXIN1–STAMBP positive link is intensified (R² = 0.9640, P < 0.0001; **Figure 5I**), while strong negative correlations simultaneously appear for FGF-19–TNFSF14 (R² = 0.6311, P = 0.0105; **Figure 5K**) and CXCL9–TWEAK, generating a conspicuous pro- versus anti-inflammatory opposition. Thus, within a hyperglycaemic milieu, formerly scattered negative regulators are consolidated into an “inhibitory cluster” that continuously counteracts the “inflammatory cluster”, driving the paradoxical state of excessive inflammation co-existing with immune incompetence characteristic of TB-DM.

**Table 1:** Positive correlations of cytokine pairs in HCs, DM, and TB-DM groups.

Groups	Cytokine pairs	r-value	P-value
HCs	AXIN1-STAMBP	0.91	5.867e-006
	CXCL5-STAMBP	0.94	3.135e-005
	AXIN1-4E-BP1	0.89	3.570e-004
	4E-BP1-STAMBP	0.89	5.097e-005
	TNFSF14-STAMBP	0.89	1.469e-004
DM	ST1A1-CASP-8	0.91	5.452e-006
	AXIN1-STAMBP	0.89	2.105e-005
	TNFSF14-STAMBP	0.86	6.196e-005
	CXCL5-STAMBP	0.86	6.875e-005
	CDCP1-CXCL10	0.81	3.551e-004
TB-DM	AXIN1-STAMBP	0.98	2.617e-006
	AXIN1- CXCL5	0.97	2.443e-005
	CXCL5-STAMBP	0.96	3.135e-005
	4E-BP1-STAMBP	0.96	5.097e-005
	TNFSF14-STAMBP	0.94	1.469e-004

**Table 2:** Negative correlations of cytokine pairs in HCs, DM, and TB-DM groups.

Groups	Cytokine pairs	r-value	P-value
HCs	CXCL5-CXCL10	-0.75	0.485
	TNFSF14-CXCL9	-0.68	0.848
	TWEAK-CDCP1	-0.65	0.403
	STAMBP-CXCL9	-0.64	0.664
	DNER-CXCL10	-0.63	0.685
DM	SCF-TRANCE	-0.66	0.010
	TWEAK-IL-6	-0.56	0.037
	SCF-FGF21	-0.46	0.098
	IFN-γ-IL-6	-0.36	0.202
	AXIN1-CXCL10	-0.35	0.214
TB-DM	FGF-19-TNFSF14	-0.79	0.011
	FGF-19-CASP-8	-0.76	0.018
	CXCL9-MCP-4	-0.75	0.020
	IFN-γ-TWEAK	-0.72	0.029
	CXCL9-TWEAK	-0.71	0.031

### 3.6 Pathway involvements and molecular mechanisms of DEPs in TB-DM compared with HCs and DM

KEGG pathway analysis of DEPs revealed significant enrichment in rheumatoid arthritis-related pathways and cytokine/Toll-like receptor interaction pathways in TB-DM vs HCs comparisons (**Table 3**). This enrichment pattern suggests that the systemic inflammation in the TB-DM state shares molecular-level commonalities with chronic autoimmune diseases and robust innate immune responses. Notably, TB-DM vs DM comparisons exhibited overlapping pathway enrichments with TB-DM vs HCs, with additional involvement of intestinal immune network for IgA production and hematopoietic cell lineage pathways (**Table 4**). These specific pathways likely reflect a more extensive remodeling of immune homeostasis triggered by MTB infection in the context of diabetes, encompassing potential alterations in the mucosal immune barrier and hematopoietic development, the implications of which warrant further investigation.

**Table 3:** Enrichment analysis of KEGG signaling pathways in HCs vs. TB-DM.

ID	Description	Gene ratio ^a^	Q value ^b^
hsa04060	Cytokine-cytokine receptor interaction	6/11	8.09937E-05
hsa04062	Chemokine signaling pathway	4/11	0.003598974
hsa05323	Rheumatoid arthritis	3/11	0.004160553
hsa04620	Toll-like receptor signaling pathway	3/11	0.004810342
hsa04623	Cytosolic DNA-sensing pathway	2/11	0.021312332

a Gene Ratio: The number of differential genes in this pathway;

b Q-value: The corrected P value. The smaller the Q-value, the higher the enrichment degree.

**Table 4:** Enrichment analysis of KEGG signaling pathways in DM vs. TB-DM.

ID	Description	Gene ratio ^a^	Q value ^b^
hsa04060	Cytokine-cytokine receptor interaction	8/12	1.82906E-07
hsa04062	Chemokine signaling pathway	5/12	0.000276045
hsa05323	Rheumatoid arthritis	3/12	0.00548554
hsa04620	Toll-like receptor signaling pathway	3/12	0.006330569
hsa04672	Intestinal immune network for IgA production	2/12	0.01855856
hsa04623	Cytosolic DNA-sensing pathway	2/12	0.021183211
hsa04640	Hematopoietic cell lineage	2/12	0.044670134

a Gene Ratio: The number of differential genes in this pathway;

b Q-value: The corrected P value. The smaller the Q-value, the higher the enrichment degree.

**Figure 6:**
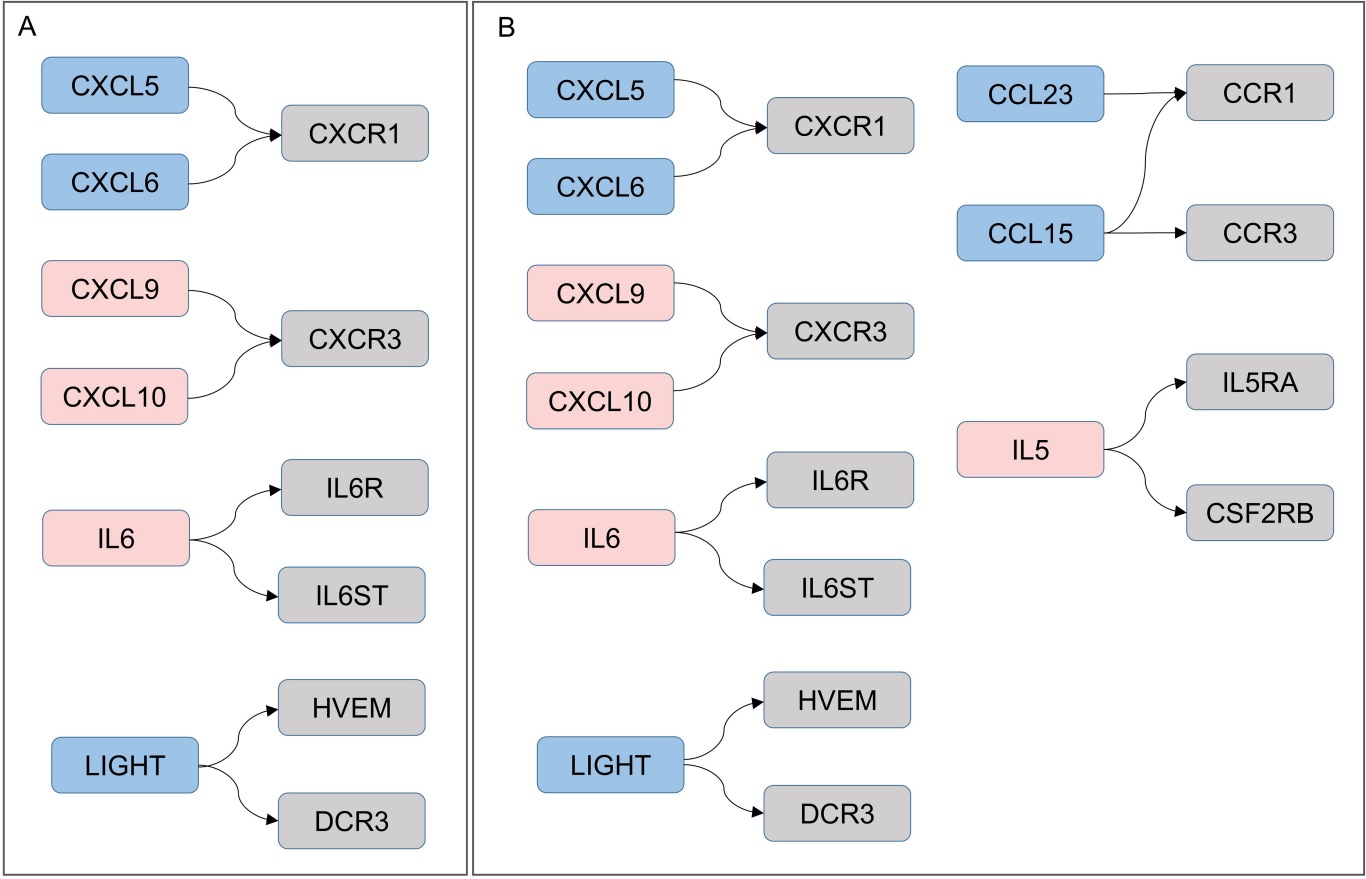
KEGG pathway enrichment of dysregulated cytokines. Cytokine-cytokine receptor interaction (hsa04060) pathway mapping: **(A)** TB-DM vs HCs: 3 proteins upregulated (red), 3 downregulated (blue). **(B)** TB-DM vs DM: 4 upregulated (red), 5 downregulated (blue). Gray rectangles denote membrane receptors.

Consistent with our previous findings linking TB-DM immunopathology to cytokine-mediated mechanisms (24), six common DEPs were identified in the hsa04060 (cytokine-cytokine receptor interaction) pathway across TB-DM vs HCs and TB-DM vs DM comparisons: CXCL5/CXCL6 (CXCR1 ligands), CXCL9/CXCL10 (CXCR3 ligands), IL-6 (IL-6R/IL6ST ligand), and LIGHT (TNFSF14; HVEM/DCR3 ligand) (**Figure 6A**). These molecules constitute a core hub network that drives Th1-type inflammation and the acute-phase response. Unique to TB-DM vs DM comparisons were CCL23 (CCR1 ligand), CCL15 (CCR3/CCR1 ligand), and IL5 (IL5RA/CSF2RB ligand) in the hsa04060 pathway (**Figure 6B**). These TB-DM-specific molecules may be linked to the activation of specific immune responses.

**Figure 7:**
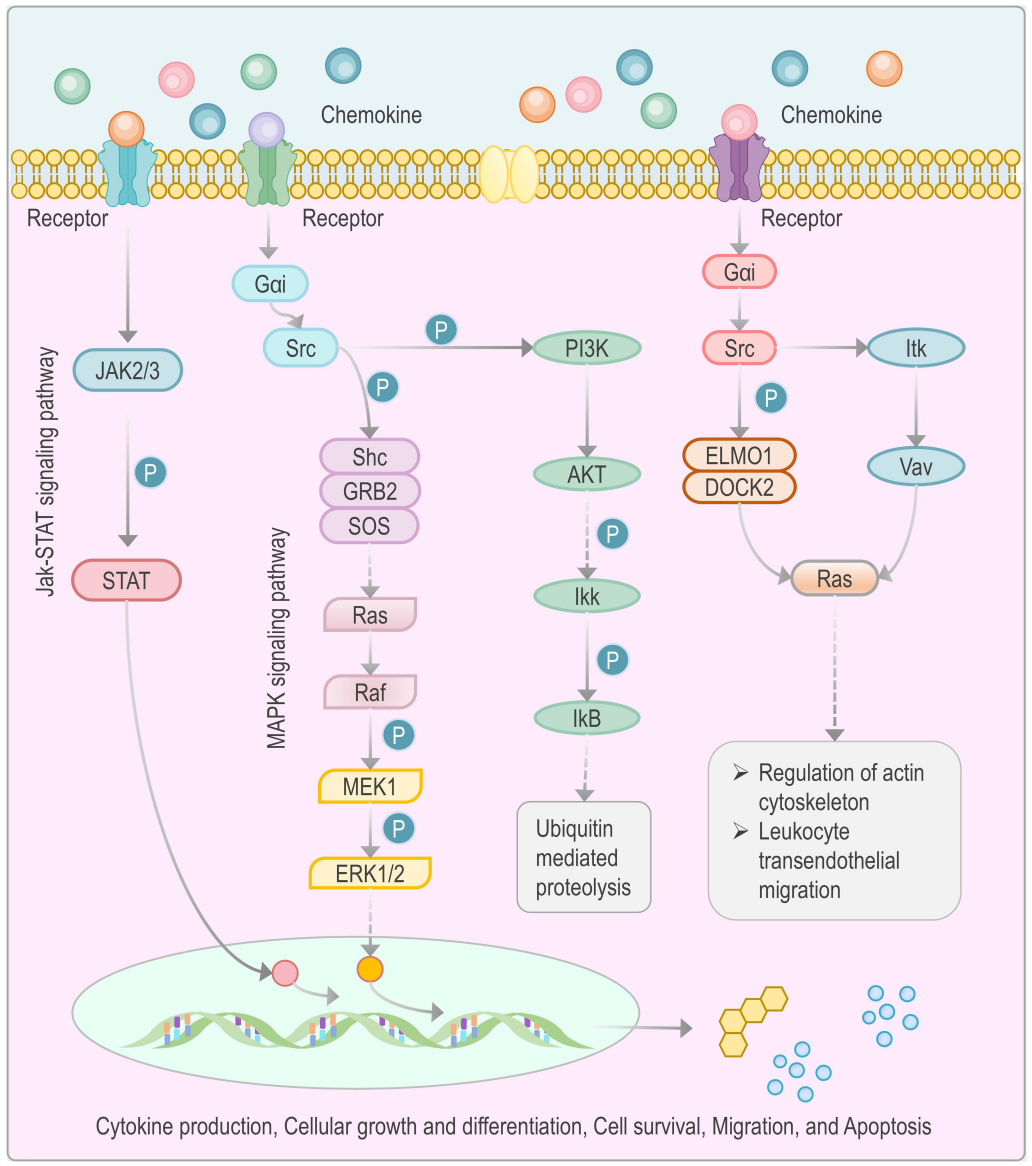
Signaling pathways involved in cytokine regulation in TB-DM comorbidity. This figure illustrates the signaling pathways implicated in cytokine regulation within the context of TB-DM comorbidity. The figure provides a detailed overview of the interactions between critical molecules within these pathways, including Gαi, Src, Ras, PI3K, AKT, and Ikk, which are involved in phosphorylation events (denoted by P) that regulate actin cytoskeleton reorganization, leukocyte transendothelial migration, and ubiquitin-mediated proteolysis. This diagram underscores the molecular mechanisms underlying the altered cytokine expression observed in TB-DM patients, as analyzed through the KEGG database pathway (hsa04062).

**Figure 8:**
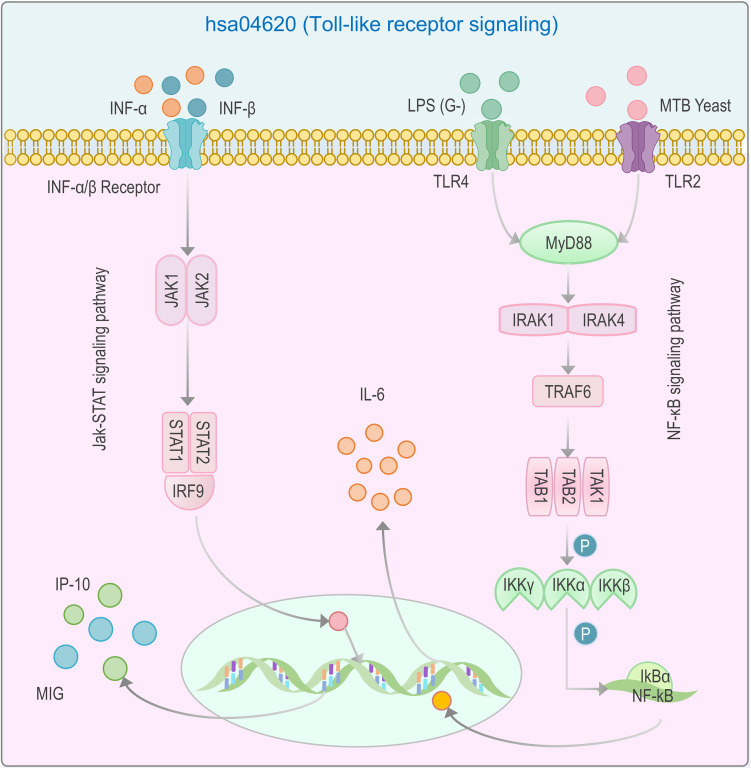
Regulation of IP-10 (CXCL10), MIG (CXCL9), and IL-6 by Jak-STAT and NF-κB signaling pathways. This diagram illustrates the activation of IP-10 (CXCL10), MIG (CXCL9), and IL-6 through the interplay of Jak-STAT and NF-κB signaling pathways in response to MTB stimuli. Jak-STAT Pathway: Initiated by INF-α/β binding to their receptors, leading to the activation of JAK1 and JAK2. These kinases phosphorylate STAT1 and STAT2, which form a complex with IRF9 to directly induce the expression of IP-10 and MIG. NF-κB Pathway: Activated via TLR2/4 recognition of MTB components, recruiting MyD88 and IRAKs (IRAK1/4), and subsequently TRAF6. This cascade activates TAK1, which phosphorylates IKKs (IKKα/β/γ), leading to IkBα degradation and NF-κB nuclear translocation. NF-κB further amplifies pro-inflammatory responses by upregulating IP-10, MIG, and IL-6. Phosphorylation events (denoted by “P”) and protein-protein interactions highlight critical regulatory steps. This dual-pathway activation underscores the coordinated immune response to MTB, with shared cytokines (e.g., IL-6) and cross-talk nodes (e.g., STAT1/NF-κB) driving inflammatory and antimicrobial activities in TB-DM comorbidity.

GO molecular function annotation demonstrated predominant involvement of these proteins in cytokine activity, cytokine receptor binding, receptor-ligand activation, and signal transducer activity (**Table 5**). This functionally confirms their role as upstream signaling ligands. Subsequent pathway mapping revealed downstream activation of Jak-STAT signaling, MAPK signaling, ubiquitin-mediated proteolysis, actin cytoskeleton regulation, and leukocyte transendothelial migration (**Figure 7**). This series of pathways clearly delineates a complete chain from ligand-receptor binding to intracellular signal transduction, ultimately leading to a functional immune phenotype. Targeted analysis of the hsa04620 (Toll-like receptor signaling) pathway further linked Jak-STAT and NF-κB signaling to IP10 (CXCL10), MIG (CXCL9), and IL-6 production (**Figure 8**). This finding is crucial, as it suggests that in TB-DM, innate immune signals triggered by pathogen recognition and cytokine-mediated inflammatory signals may converge and mutually amplify through the two core pathways, Jak-STAT and NF-κB, thereby forming and sustaining a persistent and prominent inflammatory cycle.3

**Table 5:** GO database analysis of cytokine molecular function annotation across groups.

ID	Description	Cytokines	
		HC vs TB-DM	DM vs TB-DM
GO:0005125	cytokine activity	IL6/CXCL10/CXCL9/TNFSF14/CXCL6/CXCL5	IL6/CXCL9/CXCL10/CXCL5/CXCL6/TNFSF14/CCL23/IL5
GO:0005126	cytokine receptor binding		
GO:0048018	receptor ligand activity		
GO:0030546	signaling receptor activator activity		

### 3.7 Diagnostic Performance of Single Cytokines and Their Combinations for Distinguishing TB-DM Patients from HCs and DM Patients

**Figure 9:**
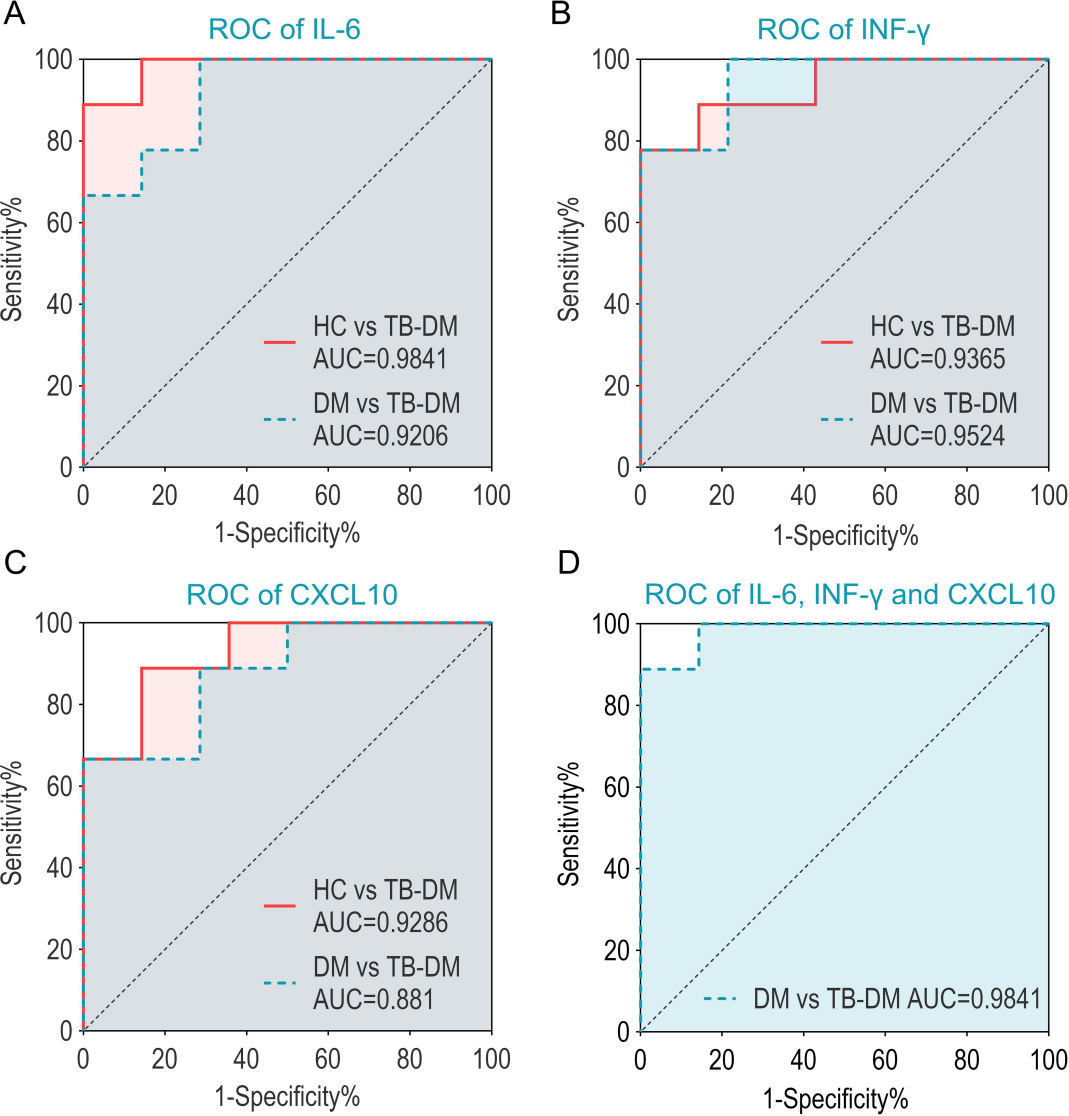
ROC curve of cytokines distinguishing TB-DM patients from HC or DM patients. This figure presents the diagnostic performance of cytokines in distinguishing TB-DM patients from HCs and DM patients, as evaluated by ROC curves. **(A)** ROC of IL-6: IL-6 exhibited significant discriminatory ability between TB-DM and HCs (AUC = 0.9841) and between TB-DM and DM (AUC = 0.9206). **(B)** ROC of IFN-γ: IFN-γ showed strong diagnostic efficacy, with AUC values of 0.9365 (TB-DM vs HCs) and 0.9524 (TB-DM vs DM). **(C)** ROC of CXCL10: CXCL10 demonstrated robust diagnostic performance, achieving AUC values of 0.9286 (TB-DM vs HCs) and 0.881 (TB-DM vs DM). **(D)** Combined ROC of IL-6, IFN-γ, and CXCL10: The combination of these three cytokines achieved an AUC of 0.9841, highlighting their synergistic diagnostic potential in distinguishing TB-DM patients from DM patients.

For single cytokine ROC curve analysis, IL-6, IFN-γ, and CXCL10 exhibited significant discriminatory abilities for distinguishing TB-DM patients from HCs and DM patients. In the TB-DM group compared to HCs, IL-6 achieved an AUC of 0.9841 (sensitivity 88.89%, specificity 92.86%) (**Figure 9A**). In the TB-DM group compared to DM, IL-6 achieved an AUC of 0.9206 (sensitivity 77.78%, specificity 85.71%) (**Figure 9A**). IFN-γ achieved an AUC of 0.9365 (sensitivity 88.89%, specificity 85.71%) in the TB-DM group compared to HCs and an AUC of 0.9524 (sensitivity 77.78%, specificity 92.86%) in the TB-DM group compared to DM (**Figure 9B**). CXCL10 achieved an AUC of 0.9286 (sensitivity 88.89%, specificity 85.71%) in the TB-DM group compared to HCs and an AUC of 0.881 (sensitivity 88.89%, specificity 71.43%) in the TB-DM group compared to DM (**Figure 9C**). Multivariable logistic regression analysis combining IL-6, IFN-γ, and CXCL10 as predictors achieved an AUC of 0.9841, sensitivity 88.89%, and specificity 92.86% (**Figure 9D**). Additionally, SCF achieved an AUC of 0.9921 (sensitivity 88.89%, specificity 92.86%) in the TB-DM group compared to HCs, but its discriminatory ability significantly decreased in the TB-DM group compared to DM (AUC = 0.7698) (**Table 6**).

**Table 6:** Sensitivity and specificity of IFN-γ, IL-6, SCF and CXCL10 cytokines in the differentiation of HCs, DM, and TB-DM patients.

Cytokine	Group	AUC	P	95%CI	Cutoff value	Sensitivity%	Specificity%
IL6	TB-DM vs HC	0.9841	0.0001	0.9436-1.000	> 3.019	88.89	92.86
	TB-DM vs DM	0.9206	0.0008	0.8117-1.000	> 3.798	77.78	85.71
IFN-γ	TB-DM vs HC	0.9365	0.0005	0.8326-1.000	> 7.210	88.89	85.71
	TB-DM vs DM	0.9524	0.0003	0.8714-1.000	> 7.589	77.78	92.86
CXCL10	TB-DM vs HC	0.9286	0.0007	0.8252-1.000	> 9.501	88.89	85.71
	TB-DM vs DM	0.881	0.0025	0.7370-1.000	> 9.537	88.89	71.43
SCF	TB-DM vs HC	0.9921	<0.0001	0.9668-1.000	< 8.627	88.89	92.86
	TB-DM vs DM	0.7698	0.0322	0.5777-0.9620	< 8.477	77.78	64.29
IL-6, IFN-γ and CXCL10	TB-DM vs DM	0.9841	0.0001	0.9436-1.000	>0.3143	88.89	92.86

## 4 Discussion

TB-DM has emerged as a significant public health challenge, with increasing incidence and severe complications. Previous studies have demonstrated that TB-DM patients exhibit enhanced immune system impairment, particularly in the context of chronic high blood sugar levels and insulin resistance (25, 26). This impairment compromises the body’s ability to combat MTB, leading to reduced immune response and increased disease progression (24). Consequently, identifying TB-DM-specific immune characteristics has become a critical area of research to better understand the disease’s pathophysiology and develop targeted therapeutic strategies.

Our comprehensive analysis of TB-DM patients revealed distinct immunological discrepancies. In DM patients, compared to HCs, NK cells showed a significant decline in absolute counts, while CD4^+^;T lymphocytes (a subset of adaptive immune cells) showed a marked increase. Notably, when disease progression advanced to TB-DM, these immunological abnormalities became more pronounced, with significant reductions in NK cells, total T lymphocytes, CD4^+^;T lymphocytes, CD8^+^;T lymphocytes and B lymphocytes across all three groups (TB-DM, DM, and HCs). This progressive and widespread reduction in immune cells may be associated with disorders of glucose metabolism, thereby leading to systemic immune dysfunction and potentially exacerbating disease progression (27, 28). However, the adaptive immune system’s partial recovery, particularly in CD4^+^;T lymphocytes, indicates a complex interplay between chronic inflammation and immune dysregulation in TB-DM.

Traditional perspectives often overlook the critical role of B lymphocytes in TB-DM, yet recent evidence highlights their importance in the disease’s pathogenesis. B lymphocytes play a pivotal role in both innate and adaptive immune responses, and their dysregulation has been implicated in the development of TB-DM (29, 30). Our study corroborated these findings by demonstrating that B lymphocytes exhibit significantly reduced absolute counts in TB-DM patients compared to HCs and DM patients, further supporting the idea that B lymphocytes are essential mediators of immune dysregulation in this disease.

Intercellular signaling plays a critical role in the recognition and activation of host immune cells (31). Immune cells regulate cytokine secretion or suppression through signaling pathways, and these cytokines may further amplify or inhibit cellular responses via positive or negative feedback mechanisms (32, 33). Previous studies suggest that TLR2- and TLR4-mediated immune responses are essential for defending against MTB invasion (34). Upon cytokine binding to TLRs, activation of the NF-κB signaling pathway stimulates DCs to secrete IL-6 and other cytokines, facilitating MTB clearance (35, 36). KEGG pathway enrichment analysis revealed enhanced IL-6 secretion via the NF-κB signaling pathway in TB-DM patients, potentially linked to hyperinflammatory responses in diabetes. This may promote neutrophil and macrophage recruitment/survival (37) and activate T-cell immunity to combat MTB. The JAK-STAT pathway was another enriched signaling axis. IFN-β binding to IFN-β receptors induces MIG (CXCL9) and IP10 (CXCL10) secretion through downstream signaling cascades. Our correlation matrix analysis demonstrated positive associations between CXCL9/CXCL10 and IFN-γ, with elevated levels of IFN-γ, CXCL9, and CXCL10 in TB-DM patients. Lande et al. reported that CXCL9 and CXCL10 are induced by IFN-β post-MTB infection (38). The CXCL9/CXCL10-CXCR3 axis exhibited significant co-regulation with the JAK-STAT pathway in TB-DM patients, suggesting its role as a metabolic-immune interface. However, individual cytokines (e.g., CXCL10) may be influenced by multiple pathways during MTB infection. NF-κB, JAK-STAT, and PI3K/Akt pathways synergistically activate CXCL10 transcription (39–41), yet only one pathway was observed in TB-DM patients, warranting further investigation into TB-DM-specific signaling mechanisms.

The identification of IL-6, IFN-γ, and CXCL10 as potential diagnostic biomarkers for TB-DM represents a significant breakthrough. These cytokines were found to exhibit marked elevation in TB-DM patients, with IL-6 levels showing a strong correlation with disease severity (42, 43). Furthermore, IL-6 has been implicated in chronic inflammation and metabolic dysregulation, particularly in the context of chronic granulomatous disease (CGD) and type 1 diabetes mellitus (T1DM) (44, 45). The increased expression of IL-6 in TB-DM patients may reflect its role in promoting immune cell migration and activation, thereby exacerbating disease progression (37, 46).

IFN-γ, another key cytokine in TB-DM, is primarily produced by activated NK cells and Th1-like T cells, and it plays a crucial role in regulating both innate and adaptive immunity (47–50). In the context of TB-DM, IFN-γ has been shown to downregulate the expression of CD4^+^;T lymphocytes and B lymphocytes, further supporting its role as a critical regulator of immune dysregulation (51, 52). Additionally, the upregulation of IFN-γ in TB-DM patients may reflect its potential role in mediating immune tolerance and resistance to MTB infection.

CXCL10, a member of the CXC family of chemotactic proteins (53), has also been implicated in the pathogenesis of TB-DM (54). Its increased expression in TB-DM patients is associated with chronic inflammation and metabolic derangements, suggesting a potential role in the disease’s progression (55). Furthermore, CXCL10 has been shown to promote the migration and activation of immune cells, particularly in the context of chronic infections (56–58). These findings highlight the importance of CXCL10 in the immune dysregulation observed in TB-DM patients (59).

The diagnostic potential of IL-6, IFN-γ, and CXCL10 was further supported by our ROC analysis. The combination of these three biomarkers achieved a diagnostic accuracy of 0.9841 (sensitivity 88.89%, specificity 92.86%), demonstrating their ability to effectively distinguish TB-DM patients from healthy controls and DM patients. This multi-dimensional approach offers a robust strategy for the early diagnosis and clinical management of TB-DM, potentially improving patient outcomes.

Despite the significant advances in understanding TB-DM’s immune characteristics, several limitations remain. First, the relatively small sample size may limit the generalizability of our findings. Second, while we have successfully identified potential biomarkers, further functional studies and animal experiments are required to clarify the causal relationship between these markers and disease progression. Additionally, the molecular mechanisms underlying the observed immune dysregulation in TB-DM remain poorly understood, necessitating further research to unravel the complex interplay between cytokines, chemokines, and immune cells in this disease. Lastly, this study did not analyze the potential impact of diabetes duration on immunological features; future prospective studies should incorporate this important variable into their design.

## 5 Conclusions

This study conducted a comprehensive analysis of the immunological features in TB-DM patients, revealing distinct immunological abnormalities characteristic of the disease. The findings indicate that TB-DM patients exhibit significantly reduced absolute counts of NK cells, CD4^+^;T cells, and B cells in their peripheral blood, with these immune cell populations becoming increasingly dysfunctional during disease progression. Based on the Olink multiplexed target-specific protein detection technology, we successfully identified IL-6, IFN-γ, and CXCL10 as potential diagnostic biomarkers for TB-DM, with their expression levels significantly elevated in TB-DM patients and correlating with disease severity. These results highlight the critical role of these cytokines in the early diagnosis and clinical management of TB-DM, offering a robust diagnostic tool for early intervention.

Through KEGG and GO pathway analysis, we identified that IL-6, IFN-γ, and CXCL10 are enriched in NF-κB, JAK-STAT, and CXC subfamily signaling pathways, suggesting their involvement in TB-DM pathogenesis through multiple immune regulation pathways. Combined with ROC curve analysis, we demonstrated that these biomarkers exhibit high sensitivity (88.89%) and specificity (92.86%), effectively distinguishing TB-DM patients from DM patients. This multi-dimensional diagnostic approach provides a highly sensitive and specific method for the precise identification of TB-DM cases, potentially improving patient outcomes.

## Data Availability

All data generated or analyzed during this study are included in this published article.

## Supplementary material

Supplementary Table 1 The detailed information of Cohort I.

Supplementary Table 2 The detailed information of Cohort II.
